# Learning to live together: mutualism between self-splicing introns and their hosts

**DOI:** 10.1186/1741-7007-9-22

**Published:** 2011-04-11

**Authors:** David R Edgell, Venkata R Chalamcharla, Marlene Belfort

**Affiliations:** 1 Department of Biochemistry, Schulich School of Medicine and Dentistry, The University of Western Ontario, London, Ontario, Canada N6A 5C1; 2 Center for Medical Sciences, Wadsworth Center, New York State Department of Health, and University at Albany, SUNY, Albany, NY 12208, USA; 3 National Cancer Institute, National Institutes of Health, Bethesda, MD 20892, USA

## Abstract

Group I and II introns can be considered as molecular parasites that interrupt protein-coding and structural RNA genes in all domains of life. They function as self-splicing ribozymes and thereby limit the phenotypic costs associated with disruption of a host gene while they act as mobile DNA elements to promote their spread within and between genomes. Once considered purely selfish DNA elements, they now seem, in the light of recent work on the molecular mechanisms regulating bacterial and phage group I and II intron dynamics, to show evidence of co-evolution with their hosts. These previously underappreciated relationships serve the co-evolving entities particularly well in times of environmental stress.

## 

One of the most intricate relationships in biology is that between a host and a parasite. Almost all organisms studied so far harbor mobile genetic elements and/or their derivatives. At the genomic level, the traditional view of mobile elements is that they provide seemingly little or no benefit to the host while parasitizing the host's cellular machinery to promote element mobility through complex molecular pathways [1,2]. The host's response to these elements is primarily defensive, as evidenced by the many forms of negative regulation that downregulate the activity of mobile elements [3-8]. The persistence of a mobile element in a given population is thus the result of a delicate balance between an excessive mutational burden on the host caused by the element's unrestricted activity, and excessive negative regulation imposed by the host on the element to limit mobility. While the relationship between host and mobile element is often viewed as a molecular arms race [9], recent experimental data argue that the relationship is more elaborate than previously appreciated.

## Mobile introns: ribozymes with baggage

One group of mobile genetic elements comprises the group I and II introns. These sequences interrupt protein-coding and structural RNA genes in all domains of life and can be considered as molecular parasites. When the gene is transcribed into RNA, the intron sequence acts as a ribozyme (an RNA with enzymatic activity), which removes the intron sequence from the primary RNA transcript, thus limiting the phenotypic cost associated with insertion of the element into a host gene and promoting their maintenance in the genome. In the case of group I and II introns, the host-parasite relationship is enriched by the fact that the introns themselves have been invaded by smaller parasitic elements - genes that encode mobility-promoting activities that enable the DNA element to move within and between genomes [10]. Thus, at least two levels of parasitism exist for mobile introns: the intron in the host gene it interrupts, and the invading gene in the intron. Collectively, the intron and its encoded mobility protein (often termed an intron-encoded protein, IEP) collaborate to form a composite mobile element that utilizes host DNA replication, recombination and repair pathways to spread [11], while the ribozyme activity ensures that it does not disrupt the function of genes into which it is inserted. Accordingly, it has become evident that there is an extraordinary degree of co-evolution among IEPs, the introns that house them, and the host organism. This review highlights several recent studies probing the interplay among self-splicing introns in bacterial and phage genomes, their genes, and their bacterial and phage hosts.

### Group I introns

**Figure 1:**
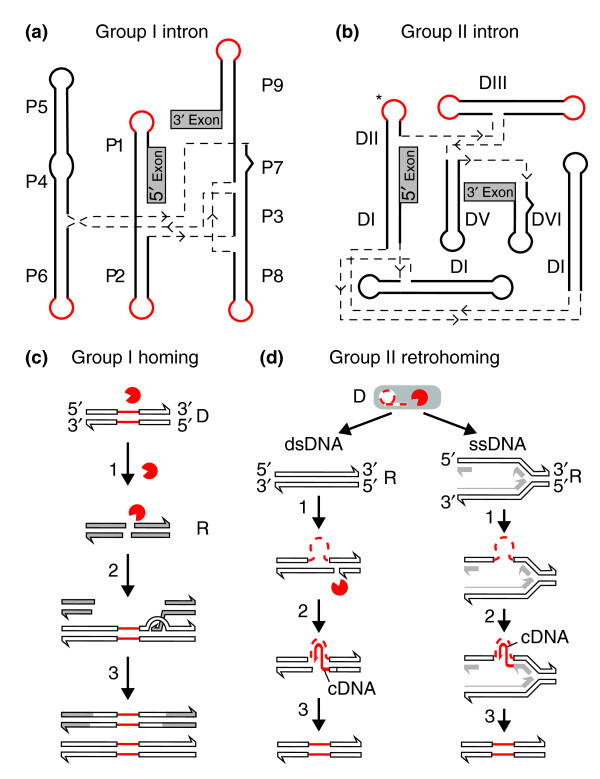
**Models of group I and group II introns and their 'homing' mechanisms**. **(a,b) **Schematic representations of (a) group I and (b) group II intron secondary structures [13,37]. In both cases, secondary structures are represented by solid lines indicating conserved stem-loop structures, named P1 to P10 for group I introns, and DI to DVI for group II introns. The positions of ORFs and other insertions are depicted by solid red lines. The asterisk (*) next to domain II of group II introns indicates bioinformatic predictions of the ORF start sites, but these remain uncharacterized. Dashed gray lines indicate joining regions of unpaired nucelotides, with arrows indicating a 5'-3' orientation. The 5' and 3' exons are indicated by grey rectangles. **(c) **Homing of a group I intron. In this DNA-based mobility pathway, the intron donor (D) expresses the intron endonuclease (red enzyme symbol) (step 1). After cleavage of the allelic intron recipient sequence (R) at the homing site (step 2) the donor and recipient engage in double-strand break (DSB) repair to generate two intron-containing alleles. **(d) **Group II intron retrohoming by means of an RNA intermediate. The intron donor (D) in this case is the spliced intron lariat RNA (dashed red line), whereas the recipient (R) can be either double-stranded DNA (dsDNA) or single-stranded DNA (ssDNA), as at a replication fork. A ribonucleoprotein complex between the RNA and the IEP catalyzes a reverse splicing (step 1). In the dsDNA pathway the IEP then cleaves the second strand to generate the primer for cDNA synthesis by the IEP, whereas in the ssDNA pathway an Okazaki fragment at the replication fork (solid gray line) acts as a primer (step 2). Second-strand cDNA synthesis followed by repair completes the retromobility reactions (step 3).

Group I introns commonly inhabit bacterial, organellar, bacteriophage and viral genomes, and the ribosomal RNA genes (rDNA) of eukaryotes, and produce a self-splicing RNA [12]. Group II introns have a similar distribution, except that they are not found in eukaryotic nuclear genes. Group I and group II introns show little primary sequence conservation, yet their RNAs each adopt characteristic secondary and tertiary structures necessary for ribozyme activity [13,14] (Figure 1a, b). Moreover, the introns can tolerate the insertion of large amounts of sequence into terminal loops of the ribozyme secondary structure with little or no effect on splicing, providing convenient 'hiding' spots for parasitic genes.

Many group I introns move by a DNA-based transposition mechanism known as 'homing'. Such introns harbor genes encoding so-called homing endonucleases, site-specific but sequence-tolerant DNA endonucleases that introduce double-strand breaks (DSBs) in cognate alleles that lack the intron, initiating intron mobility via a DSB-repair process [11] (Figure 1c). The outcome of a homing event is the unidirectional movement of the intron and endonuclease open reading frame (ORF) to an unoccupied allele, leaving a copy of the intron in its original location (Figure 1c). Group I introns can also harbor other 'baggage'. Many group I introns in organellar genomes encode maturases - proteins that help promote intron splicing by a variety of mechanisms [15,16]. Some maturases also function *in trans *to promote splicing of other group I introns in the same genome [17,18]. Interestingly, many maturases characterized so far are degenerate or bifunctional homing endonucleases of the LAGLIDADG class - so named for their conserved sequence motif - that have acquired an RNA chaperone activity independent of their DNA endonuclease activity [19,20]. Group I introns can also harbor ORFs unrelated to mobility or splicing [21,22], as exemplified by the astonishing case of an approximately 18-kilobase-long intron inserted in the mitochondrial *ND5 *gene of the mushroom coral *Discosoma *that encodes 15 mitochondrial genes in the P8 loop of the intron [23,24]. Interestingly, these 15 genes include both the small and large subunit rRNA genes and the *cox1 *gene, which is interrupted by another self-splicing group I intron.

Some bacterial group I introns have been invaded by mobile elements other than those that encode homing endonucleases. Notable among these are the chimeric intron/insertion sequence (IS) elements (IStrons) of *Clostridium *that contain an *IS605*-like element inserted at the 3' end of the intron [25]. It is not known, however, whether the chimeric intron/IS element is mobilized by the *IS605 *machinery. Intriguingly, another unusual clostridial group I intron arrangement was recently found by a bioinformatic search for riboswitches [26], RNA structural elements that control gene expression through alternative secondary structures in response to binding of secondary metabolites. In this case, the tandem riboswitch/intron lies in the upstream region of a putative virulence factor gene, and sensing of cyclic di-guanosyl-5'-monophosphate by the riboswitch controls choice of the 3' splice junction by the intron to modulate expression of the virulence factor.

**Figure 2:**
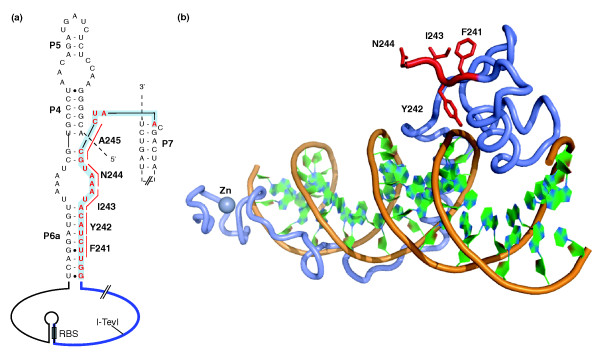
**Overlap of the I-TevI ORF with core *td *intron sequence**. **(a) **Secondary structure of the relevant portion of the *td *intron from phage T4 [27], labeled as in Figure 1. The I-TevI ORF is located in the P6 loop (solid blue line), but extends into the core *td *structure, as indicated by the last 20 nucleotides (colored red) of the I-TevI ORF, which contribute to P6a and P7. Short red lines to the side of these nucleotides indicate codons corresponding to the five carboxy-terminal amino acids of I-TevI (F241 to A245). The RNA hairpin that sequesters the I-TevI ribosome-binding site (RBS) is indicated in the P6 loop [55,59]. **(b) **Co-crystal structure of the I-TevI 130C DNA-binding domain with intronless DNA substrate [47], modified from PDB 1T2T using PyMol. The amino acids corresponding to the region of overlap with the *td *intron sequence are shown as red sticks, with the remainder of the I-TevI protein colored blue. The DNA strand backbones are in yellow with the bases in green. Note that the carboxy-terminal alanine (A245) was not present in the I-TevI structure. The zinc ion coordinated by the I-TevI zinc finger is shown as a blue sphere.

While many ORFs embedded within group I introns are entirely located in loop regions, a surprising number of ORFs extend beyond peripheral loops to contribute nucleotides to more distant regions of the intron that form key structural elements needed for splicing [27]. The extent of the contribution of ORF sequence to ribozyme structural elements varies depending on the particular intron-ORF arrangement. For instance, in the well-studied bacteriophage T4 *td *intron, the 3' end of an ORF called I-TevI (which encodes a homing endonuclease of the GIY-YIG type, again named for a conserved sequence motif) contributes 20 nucleotides that form part of the P6a, P6.0 and P7 structures that are essential for splicing of the intron [28] (Figure 2a). In other cases, the extent of the overlap is greater, involving the 5' end as well as the 3' end of the endonuclease ORF (Table 1). It should be noted, however, that the extent of overlap noted is based on predictions of endonuclease ORFs, and it is possible that many cases of extensive overlap result from incorrect bioinformatic identification of the 5' and 3' ends of endonuclease genes. Regardless of this, the presence of an endonuclease ORF within a highly structured RNA molecule poses a number of fascinating evolutionary and functional questions. Specifically, how did composite mobile introns evolve, and what are the functional consequences of translation of endonuclease ORFs from within such highly structured RNA molecules?

**Table 1:** Examples of ORF overlap with core group I intron sequences

Organism	Host gene interrupted	Endonuclease family	Insertion site within intron	Overlap with intron (nucleotides)	Structural element overlap
*Bacillus thuringiensis *sup. *pakistani *[90]	*nrdF*	GIY-YIG	P6a	56	P6a/P7/P7.1/7.1a
*Bacillus *phage SPO1 [91]	DNA polymerase	HNH	P8	9	P8
*Synechococcus lividus *[92]	rDNA LSU	LAGLIDADG	P8	81	P6/P7/P3/P8/P9.0/P9
*Synechocystis *PCC 6803 [93]	tRNA^fmet^	PD-(D/E)-XK	P1	61	P1/P2
*Physarum polycephalum *[94]	rDNA LSU (nucleus)	His-Cys box	P1	0	None

### Group II introns

Similar questions can be asked regarding group II introns, which are found in similar niches to group I introns, but not in nuclear genomes [14]. Group II introns all have a common ribozyme structure consisting of six helical domains (Figure 1b) [29,30]. Their mobility-promoting IEPs are typically encoded within domain IV, and the introns move via an RNA-based mechanism known as 'retrohoming'. Unlike their group I intron counterparts, the group II IEPs are multifunctional proteins containing maturase (X), reverse transcriptase (RT) and DNA-binding (D) functions in addition to DNA endonuclease (En) activity. The maturase activity facilitates intron splicing by stabilizing the catalytically active RNA conformation, while the RT, D and En functions aid in RNA-based mobility pathways. In this type of movement, the spliced intron lariat RNA invades either double- or single-stranded DNA ((Figure 1d). As well as retrohoming to allelic target sites, group II introns can transpose to non-allelic sites [11,14].

Group II introns can also be invaded by elements encoding proteins other than the multifunctional IEPs. These elements include, but are not limited to, simple LAGLIDADG endonuclease ORF insertions in domain III [31,32]. Another arrangement produces the so-called twintrons, in which a group II intron has inserted into another group II intron, as in the case of the *psb *locus in *Euglena gracilis *chloroplast DNA and the TelI introns in the cyanobacterium *Thermosynechococcus elongatus *[33-35]. Whereas some insertions functionally 'split' the group II intron and interfere with intron splicing, others, such as some eukaryotic organellar introns, allow *trans*-splicing [36].

Recent crystallographic studies on the ribozyme structure of the *Oceanobacillus iheyensis *group II intron Oi5γ revealed that coaxial stacking of domain IV with its neighboring domain III projects domain IV away from the ribozyme core, probably preventing nonproductive interactions of the IEP coding sequence with the ribozyme core [37]. Likewise, the positioning of domains II and III away from the ribozyme suggests that they can accommodate additional sequence [29,38]. Although domains II, III and IV may enhance splicing efficiency, they are not strictly required for catalysis, making them hospitable sites for invasive elements. In bacteria, IEPs are encoded entirely within loops of their host group II introns, and possess regulatory features such as promoters and ribosome-binding sites that are distinct from those that control expression of the host gene in which the intron resides [39]. In contrast, in organellar genomes, ORFs embedded within group II introns are regulated by promoters in the upstream exons [40,41]. Thus, the intron ORFs are initially translated as fusion proteins with the 5' exon and require subsequent proteolytic processing [40,41].

## Visitors make themselves at home: core creep

Many lines of evidence suggest that both group I and group II introns were ancestrally ORF-less, only to be invaded multiple independent times to create composite mobile elements. Notably, ORFs are located at different positions within introns; similar introns contain different ORFs; and similar ORFs occur in divergent introns. Several hypotheses have been put forward to explain the origin and evolution of mobile introns [42-46], with each hypothesis relying on illegitimate recombination pathways to create a composite mobile intron consisting of intron and endonuclease ORF. These hypotheses do not, however, address the evolutionary and functional ramifications of the overlap of protein ORFs with key structural elements of their host introns. Also worth considering are the multiple selective pressures on ORFs that extend into the ribozyme core: the ORF sequence must evolve in such a way so as not to accumulate substitutions that adversely affect endonuclease activity (and hence affect the spread and retention of the intron in populations); while at the same time it must co-evolve with disparate regions of the intron to ensure that secondary structure elements necessary for splicing are maintained by compensatory base-pairing interactions.

We propose an alternative scenario for invasion of introns by ORFs in which ORF insertion into peripheral loops of the introns was favored, such that the ORF sequence did not overlap with core intron sequences, thus limiting any phenotypic cost associated with reduced intron splicing. This scenario also avoids the requirement that the invading ORF would have to contain exactly the same nucleotides as it was replacing in order to maintain the crucial base pairing required for intron folding. Instead, we argue that the current overlap of intron ORFs with core intron sequences occurred after invasion by a process we term 'core creep'. Essentially, this is an extension of the coding region by mutation of an existing termination codon into one specifying an amino acid, so that the ORF is extended until the next occurrence of an in-frame termination codon. For intron-encoded ORFs that underwent core creep, the next termination codon could lie within ribozyme core sequences, resulting in the overlap exhibited in many intron-ORF arrangements. Similarly, selection of an alternative initiation codon can account for the observation that the 5' ends of some endonuclease ORFs include intron core sequences.

Importantly, this hypothesis gives rise to a number of testable predictions. First, the length of the 5' or 3' extension should be variable for each independent case of endonuclease invasion, and the position of the initiation or termination codon should be influenced by the GC content of the intron because termination and initiation codons are slightly more AT rich than GC rich and the GC content of the intron will therefore influence the probability of mutation of a sense codon into a nonsense (stop) codon. The second prediction is that the sequence at the 5' or 3' ends of an endonuclease ORF that extends into the intron may not be essential for endonuclease function. In the case of I-TevI, the 20 nucleotides that extend into the *td *intron encode 6 amino acids on the carboxy-terminal end of I-TevI (out of a total 245 amino acids). In the crystal structure of the I-TevI DNA-binding domain bound to DNA representing either its homing target site or its target operator site, only one of the carboxy-terminal residues (Tyr242) makes a hydrogen bond to the phosphate backbone of the DNA substrate, clearly not providing any specificity to the interaction of I-TevI with its DNA substrate [47]. Bioinformatic searches with the I-TevI amino acid sequence also show that the carboxyl terminus is variable in length and composition (DRE, unpublished observations), implying that it is not critical for function.

## Don't bite the hand that feeds you: translational regulation of intron ORFs

The successful spread and retention of mobile introns depends on expression of the mobility-promoting protein from within the intron, and on accurate splicing-out of the introns from the flanking exon sequences. For most group I introns, mobility and splicing are independent of each other, whereas for group II introns, and some organellar group I introns, these processes are not mutually exclusive. In these cases, translation of the IEP from the pre-splicing intron transcript is necessary for splicing because the IEP acts as an RNA maturase, in addition to facilitating mobility (reviewed in [14]). Furthermore, for group II introns, the spliced-out ribozyme is the agent of mobility, integrating into the DNA target [48] (Figure 1d). Thus, translation of intron-encoded proteins must be carefully orchestrated so as not to interfere with intron-splicing pathways, and recent studies have revealed that diverse mechanisms are employed to regulate ORF expression and intron splicing.

One potential barrier to efficient intron splicing in bacterial and organellar genomes is the coupled nature of transcription and translation, which raises the possibility that ribosomes translating the RNA transcript could encounter the 5' exon-intron junction before the 3' splice site of the intron is transcribed, thus preventing the folding of critical intron structures and recognition of the correct splice sites by the intron. Ironically, a number of studies with bacterial group I introns have shown that translation of the exon upstream of the 5' splice site is necessary for efficient splicing, probably because a ribosome at this position acts as a 'chaperone' to prevent nonproductive interactions between exon and intron sequences that would disrupt the intron-folding pathway [49-51]. Most group I introns also have an in-frame stop codon positioned immediately downstream of the 5' exon-intron junction to prevent ribosome entry into the structured intron RNA. Ribosome entry into the intron core could also occur as a result of translation events that initiate at ORFs embedded within the intron. The various approaches to downregulating the translation of intron-encoded ORFs in prokaryotic genomes include the presence of non-AUG initiation codons and non-consensus ribosome-binding sites [52,53].

More complicated types of regulation are implied by numerous examples of ribosome-binding sites in introns that are sequestered by RNA secondary structure [54-58]. Mutational analysis of one such RNA secondary structure that regulates translation of the I-TevI homing endonuclease revealed a pronounced splicing defect resulting from ribosome occupancy of intron sequences that form the crucial structures necessary for splicing [59]. A different strategy of regulating translation from within a bacterial group II intron has been revealed by detailed biochemical studies of the LtrA protein encoded within the LI.LtrB group II intron of *Lactococcus lactis *[60,61] . LtrA binds with high affinity to the intron RNA, occluding the Shine-Dalgarno sequence necessary for translation of LtrA, and presumably limiting access of the ribosome to structured regions of the group II ribozyme.

Structured group I introns interrupt the nuclear rDNA of many eukaryotes, in which coupled transcription and translation is not an issue, but they nonetheless face a different set of problems connected with intron-encoded ORFs. The well-studied group I introns in rDNA genes in the slime mold *Didymium *[62] contain ORFs known as I-DirI and I-DirII. On transcription of the rDNA by polymerase I (Pol I), these ORFs are embedded within a transcript that is not able to be translated. How then can these proteins get expressed? In the case of I-DirII, the ORF is in the antisense orientation relative to the rDNA transcription unit, and expression of I-DirII is driven by its own RNA polymerase II promoter, followed by removal of a spliceosomal intron and addition of a poly(A) tail [63]. I-DirI is in the same orientation as the Pol I rRNA transcript [64], and has a more complicated expression mechanism. Maturation of a transcript competent for translation involves excision of an unusual branching ribozyme (known as DiGIR1) from the 5' end of the intron that generates a 2'-5' cap structure [65]. This is followed by processing of the 3' end and addition of a poly(A) tail. These types of regulation imply an extraordinary degree of co-evolution between intron, IEP and host gene that can best be explained by selective pressures to regulate intron splicing and ORF expression so as to not impart any phenotypic cost associated with expression of the (often essential) interrupted host gene.

As noted earlier, many proteins encoded within introns in organellar genomes are initially translated as fusions with upstream exon sequences, requiring subsequent proteolytic processing to provide an active protein with an amino terminus in domain IV [40,41,66]. Little is known about the molecular machinery required for this process, due in part to the technically demanding nature of organellar biology, but this arrangement creates opportunities for regulatory cross-talk between translation of the upstream exon and splicing, in ways that need to be determined experimentally.

## Host factors that regulate mobility: mutualism or repression?

Host-encoded proteins function to stimulate the splicing of group I and II introns. In the case of group II introns, host-function-assisted splicing is also crucial for mobility, as the spliced intron RNA is an active intermediate in the mobility pathway [67]. Detailed biochemical and structural studies have shown that host proteins function as maturases to stabilize the active group I or II RNA structure, as chaperones to resolve 'kinetic traps' that limit the rate of RNA folding, or as transporters to ensure the level of Mg^2+ ^is sufficient for efficient folding and splicing. The requirement for host-encoded proteins is especially evident for many organellar group II introns: at least 14 nuclear gene products promote efficient splicing of the two group II introns in the chloroplast-encoded *psaA *gene of *Chlamydomonas reinhardtii *[68,69]. Another example of host-facilitated intron splicing involves the Mg^2+ ^transporter Mrs2p, and the chaperone activity of three DEAD-box proteins, Mss116p, Ded1p and Cyt19p, to promote group II intron splicing in the mitochondria of fungi [70-74].

In terms of mobility, the primary response of a host genome to the presence of mobile elements is repressive, as unregulated mobile element activity will lead to an unbearable mutational load. In recent years, a number of studies have uncovered host proteins that downregulate the activities of mobile introns, many of which (not unexpectedly) are involved in aspects of RNA processing. These proteins include RNase E and RNase I, which negatively regulate group II intron mobility by reducing the steady-state level of intron RNA [75-77]. At the same time, a greater appreciation of the intricate relationships between introns and host factors that stimulate their mobility has arisen from observations that group I and II introns are obligately dependent on host-encoded functions to complete the mobility process. In the case of group I introns, the involvement of the intron-encoded homing endonuclease in mobility is limited to the introduction of a DSB (or of a single-strand nick [78,79], depending on the endonuclease) in cognate alleles that lack the intron. Completion of the mobility process requires host-encoded proteins that function in DNA recombination, replication and repair pathways [80-82]. Likewise, the retromobility pathways of group II introns are dependent on host machinery, as illustrated by the Ll.LtrB intron in *Escherichia coli *where host factors, including the major replicative polymerase Pol III, repair polymerases Pol II, Pol IV and Pol V, the endonuclease RNase H1, and DNA ligase, all function to complete a retromobility event [75]. Thus, the two intron types exploit different aspects of the host's nucleic acid transaction pathways.

## Molecular lifeboats - abandon ship!

**Figure 3:**
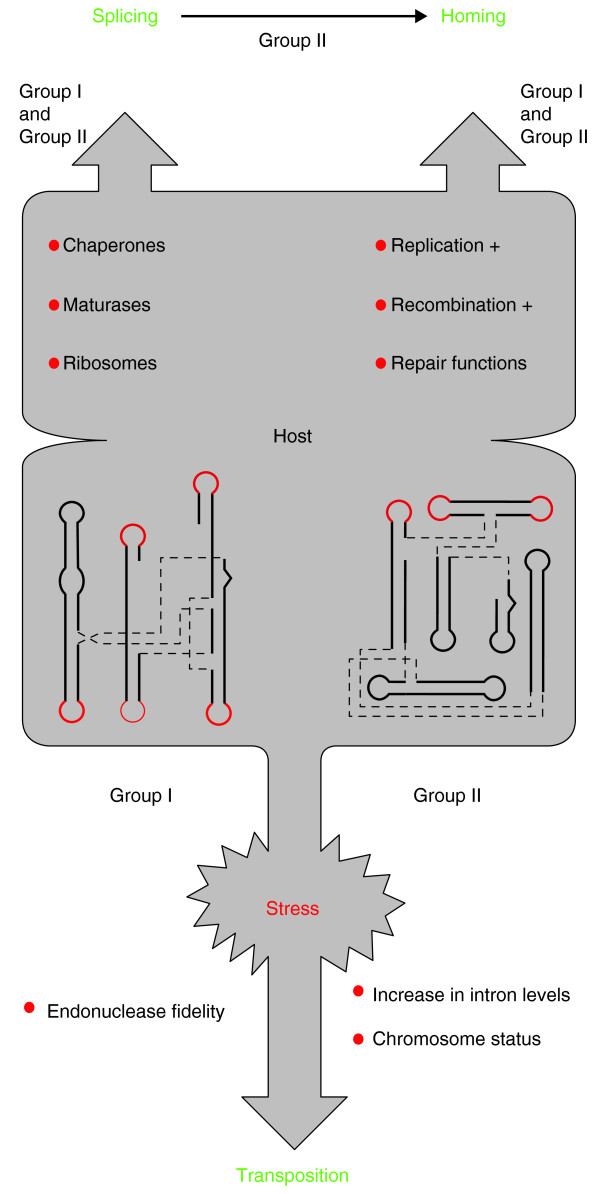
**Model for intron-host interactions**. The top half of the figure indicates that RNA chaperones and, sometimes, maturases and/or ribosomes are required to facilitate splicing of group I and group II introns, whereas replication, recombination and repair functions are necesary for homing of these elements in a host cell in a well balanced growth environment [80,95]. Splicing of group II introns is, in turn, required for their mobility [15]. The bottom of the figure indicates how mobile introns respond to stress conditions in their host cell. For group I introns, oxidative stress results in group I endonuclease substrate infidelity, allowing transposition of the intron to sites with less sequence similarity than the normal allelic target [83]. For group II introns, nutritional stress increases their rate of transcription, thus raising the level of intron RNA, and also alters the disposition of the nucleoid (the bacterial DNA) in ways that favor retrotransposition; together these changes result in a burst of retrotransposition of group II introns in response to the stress [77].

Up to this point we have considered the dynamic interplay between introns, their intramolecular inhabitants and their hosts, without considering evolutionary and environmental factors that might influence these partnerships. One traditional view of introns is that they are purely selfish DNA elements, imparting neither benefit nor burden to the host genome in which they reside. Recent evidence, however, has forced a re-evaluation of this viewpoint, particularly in the light of experimental data showing that introns can mobilize in response to stress-induced conditions [77,83], as has been demonstrated for other mobile elements [84,85]. These data raise the fascinating possibility that introns are 'plugged' into host metabolic pathways in ways that control and favor intron dissemination in times of environmental stress (Figure 3).

For instance, the group I intron endonuclease I-TevI (described in Figure 2) is subject to post-translational control under oxidative stress [83]. A zinc finger in an interdomain linker of I-TevI is redox-sensitive, and under oxidizing conditions is disrupted by loss of the zinc ion, leading to spurious DNA cleavage and intron movement to sites less similar in sequence to its usual allelic target. Reducing conditions restore zinc-finger function, cleavage and homing fidelity. This redox-responsive zinc-ion cycling suggests a mechanism for rapid, regulated group I intron dispersal under conditions of oxidative stress (Figure 3).

Group II introns respond to metabolic stress with a burst of retrotransposition to new sites by a mechanism different from that used by group I introns. Retrotransposition of the lactococcal Ll.LtrB group II intron in *E. coli *is not only regulated by RNase E [76], but is also wired into the cell's global genetic circuitry via the two small-molecule effectors ppGpp and cAMP [77]. These global regulators, which are elevated during the 'stringent response' to amino acid starvation and upon glucose starvation, respectively, stimulate retrotransposition. Whereas the RNase E effect is mediated at the level of the invading intron RNA, the global regulators are proposed to act by stalling of chromosomal replication forks and/or altering the transcriptional state of the nucleoid (that is, chromosome status), both of which might provide introns with access to the genome (Figure 3).

Clearly, the mechanisms whereby these variant introns respond to oxidative and nutritional stresses in order to disseminate are different, but with similar outcomes - the 'abandoning of ship' for more hospitable genomic environs. For the group I intron, the mobility machinery itself, the intron endonuclease I-TevI, transduces the signal [83]. Whereas intron levels can also affect retrotransposition of the group II intron [76], the signal can, in addition, be transmitted through changes in macromolecular disposition of the host [77]. One (yet to be demonstrated) evolutionary consequence of this coupling between sensing of environmental conditions and intron dissemination is the potential to generate genetic novelties that are useful to the cell under stress. A documented mechanism for introns to generate genetic diversity is through alternative splicing pathways [86,87]. In bacteriophage Twort, which infects *Staphylococcus aureus*, the ORF *orf182 *is interrupted by three similar group I introns, and analysis of spliced products revealed that some transcripts lack one exon, suggestive of programmed exon skipping [88]. Similarly, *trans*-splicing between highly similar group II introns in organellar genomes also has the potential to generate novel transcripts [89]. It is tempting to speculate that these alternative splicing events can be regulated by the host to generate novel protein products under specific cellular conditions.

## Evolving perceptions about self-splicing introns

Recent results have challenged our perceptions regarding self-splicing introns, from the notion that they represent simple genomic parasites imparting neither cost nor benefit to the host genome, to that of sophisticated mobile DNA elements fully integrated into host-cell metabolism in ways that could be viewed as molecular mutualism. Host organisms devote considerable resources, whether by design or accident, to both positively and negatively influence intron behavior, and elucidating the molecular basis of host-factor involvement in the regulation of intron splicing and mobility is one area ripe for future investigation. In particular, the mechanism underlying the processing of intron ORFs that are initially translated as fusion proteins with upstream exons in organellar introns represents an obvious gap in our knowledge, but this is a technically daunting problem to address. However, it is questions of an evolutionary slant that will challenge intronologists for years to come. Foremost among these is the possibility that introns could provide some benefit to hosts by generating genetic diversity as a consequence of transposition events brought on by cellular stress.
